# Comparative numerical finite element analysis of tooth–implant- versus implant–implant-supported three-unit fixed prostheses under oblique loading

**DOI:** 10.1007/s10266-026-01336-0

**Published:** 2026-02-04

**Authors:** Hussain D. Alsayed, Islam G. Shahin, Noha M. Anany, Mohamed I. El-Anwar, Tarek M. Elshazly, Abdulaziz Alhotan

**Affiliations:** 1 Department of Prosthetic Dental Sciences, College of Dentistry, King Saud University, Riyadh, Saudi Arabia; 2 Faculty of Dentistry, Ain Shams University, Cairo, Egypt; 3 Private Clinic, Abu Dhabi, UAE; 4 Department of Mechanical Engineering, National Research Centre, Giza, Egypt; 5 Dental School, Oral Technology, University Hospital Bonn, Bonn, Germany; 6 Department of Dental Health, College of Applied Medical Sciences, King Saud University, Riyadh, Saudi Arabia

**Keywords:** Dental implant, Zirconium, Polyetherketoneketone, Biomechanics, Stress

## Abstract

To investigate the biomechanical performance of fixed dental prostheses supported by either tooth–implant or implant–implant configurations, fabricated from zirconia or polyetherketoneketone (PEKK), under oblique loading using three-dimensional finite element analysis (FEA). Three mandibular posterior models were developed: M1 (implant at the first molar and natural tooth at the first premolar), M2 (implant at the first premolar and natural tooth at the first molar), and M3 (implants at both the first premolar and first molar). Each model was restored with a three-unit prosthesis fabricated from either zirconia or PEKK. The digital workflow included anatomical segmentation using Mimics software, refinement of bone and soft tissues with 3-Matic software, and integration of implants, abutments, and prosthetic components, followed by meshing and FEA using ANSYS software. All models were subjected to oblique loading of 50 N at 30° and 45°, and von Mises stress and deformation were recorded. M3 exhibited the most favorable biomechanical behavior, showing the lowest von Mises stress across almost all components. The hybrid tooth–implant models (M1 and M2) showed higher stress concentrations, particularly M2 with PEKK (loading angle 45°), which recorded the highest implant stress (312 MPa) and the greatest deformation (75 µm). The highest screw stress was recorded in M1 with PEKK, reaching 483 MPa. Zirconia exhibited higher stress within the prosthesis, but lower deformation, whereas PEKK resulted in greater deformation in the tooth and periodontal ligament (PDL). Implant–implant-supported prostheses showed the most favorable biomechanical behavior under oblique loading. Tooth–implant models, especially with anterior implant placement, exhibited higher stress and deformation. Zirconia offered greater rigidity and dimensional stability, while PEKK provided better stress dissipation but required careful design to manage its flexibility.

## Introduction

Dental implant therapy is widely recognized for its high long-term clinical success rates and its ability to address a broad range of functional and esthetic challenges associated with edentulism, ultimately improving patients’ quality of life [1–3]. Despite these favorable outcomes, the long-term survival and success of dental implants remain heavily dependent on several biomechanical factors. These include the quality and quantity of the supporting bone in edentulous sites, the number and spatial configuration of implants, the design and material of the prosthetic superstructure, and individual variations in occlusal forces and masticatory dynamics [1, 4–7].

In cases of partial edentulism, hybrid tooth–implant-supported prostheses are often indicated for clinical situations such as distal extension edentulism, severe alveolar ridge defects, or anatomical and economic limitations [8, 9]. However, biomechanical concerns arise from the differing mobility characteristics of osseointegrated implants and natural teeth, potentially leading to stress concentration and component failure [10, 11]. Nonetheless, longitudinal clinical studies have demonstrated the viability of such prostheses, with no significant negative biological consequences when proper protocols are followed [12–14]. To minimize complications, clinical guidelines generally advise against the use of short implants, endodontically treated abutments, and poor-quality bone, while recommending rigid connectors and definitive cementation [9]. Even with such precautions, complications such as implant failure, abutment damage, and prosthetic issues may still occur, emphasizing the importance of careful case selection and thorough biomechanical evaluation.

While vertical forces dominate during mastication, lateral or oblique loads also play a significant role in the biomechanical performance of oral structures and implant-supported prostheses. These non-axial forces, which arise during parafunctional habits, or even during unilateral chewing and eccentric mandibular movements, can induce higher stress concentrations in both the implant components and the surrounding bone compared to axial loading. Lateral forces are particularly critical because they generate bending moments that the implant–bone interface is less adapted to withstand, potentially leading to micro-movements, crestal bone loss, or mechanical complications such as screw loosening, prosthetic fracture, or implant fatigue failure [15, 16]. In natural dentition, PDL helps to dissipate such forces, but this adaptive capacity is absent in osseointegrated implants, making the system more vulnerable under lateral stress [17]. Therefore, evaluating the effects of oblique loading is essential for optimizing prosthetic designs and ensuring long-term implant stability, particularly in hybrid tooth–implant systems where differential mobility may exacerbate stress transmission.

Finite element analysis (FEA) has become a widely accepted computational tool for investigating the biomechanical behavior of dental implants across various clinical conditions [4, 5, 18]. Through the discretization of complex structures into finite elements and nodes, FEA allows for the simulation of mechanical responses under defined loading conditions, offering insight into stress and strain distribution within the implant system and surrounding bone [19]. This method aids in optimizing the prosthetic designs and materials, thereby contributing to improved implant performance and longevity [5, 20–22].

In light of the ongoing debate regarding the biomechanical behavior of hybrid tooth–implant-supported prosthesis, the aim of the present study is to utilize FEA to investigate the effects of oblique loading on three distinct prosthetic configurations: two hybrid models and one fully implant-supported model as a control. To further explore the influence of prosthetic material properties under lateral forces, each configuration was assessed using two different materials (Zirconia and PEKK).

## Materials and methods

### Study design

This FEA study evaluated the biomechanical response of three posterior mandibular prosthetic configurations. Two models, M1 and M2, represented hybrid prostheses supported by one implant and one natural tooth, while the third model, M3, featured a fully implant-supported prosthesis. In M1, the implant was placed at the first molar region (distal abutment) with the first premolar serving as the mesial tooth abutment, whereas in M2, the implant was positioned at the first premolar site (mesial abutment) with the first molar acting as the distal tooth abutment. Model M3 included two implants placed at the first premolar and first molar sites to support a three-unit bridge with a pontic replacing the second premolar. Each configuration was restored using either monolithic zirconia or polyetherketoneketone (PEKK), resulting in six experimental models: M1-Z, M2-Z, and M3-Z for the zirconia group, and M1-P, M2-P, and M3-P for the PEKK group.

### Geometric model construction

Ethical approval was obtained from the Research Ethics Committee at the Faculty of Dentistry, Ain Shams University, Egypt (FDASU-RecIE121904). A cone beam computed tomography (CBCT) scan of the mandible of an edentulous patient was imported into Mimics 14 (Materialise, Leuven, Belgium) for segmentation. After segmentation, the spongy cancellous core was enclosed by an approximately 2-mm thick cortical shell. The processed geometry was exported as an STL file and refined in 3-Matic 7.0 (Materialise, Leuven, Belgium) to correct any defects and apply smoothing algorithms. A uniform 0.3 mm PDL and a 1 mm oral mucosa layer were created using offset functions of the software. Digitally prepared lower first premolar and molar models (Digimation, St Rose, Louisiana, USA) were designed to reflect standard clinical guidelines and ensure proper prosthesis fit. The natural tooth preparation was modeled with a total occlusal convergence (taper) of approximately 6°, a preparation height of 4 mm, and a chamfer finish line at the cervical margin. A tioLogic implant system (Dentaurum, Ispringen, Germany) with a 9.0 mm length and 3.7 mm diameter (commonly clinically used dimensions) was imported into the software environment. The implant assembly included the fixture, abutment, and abutment screw. The assembled components for each configuration were imported into Exocad DentalCAD (Exocad, Darmstadt, Germany) to construct the suprastructure. Finalized models were then exported in IGES format to SolidWorks V2014 (Dassault Systèmes, Paris, France) for solid geometry repair and conversion.

### Finite element meshing

**Fig. 1:**
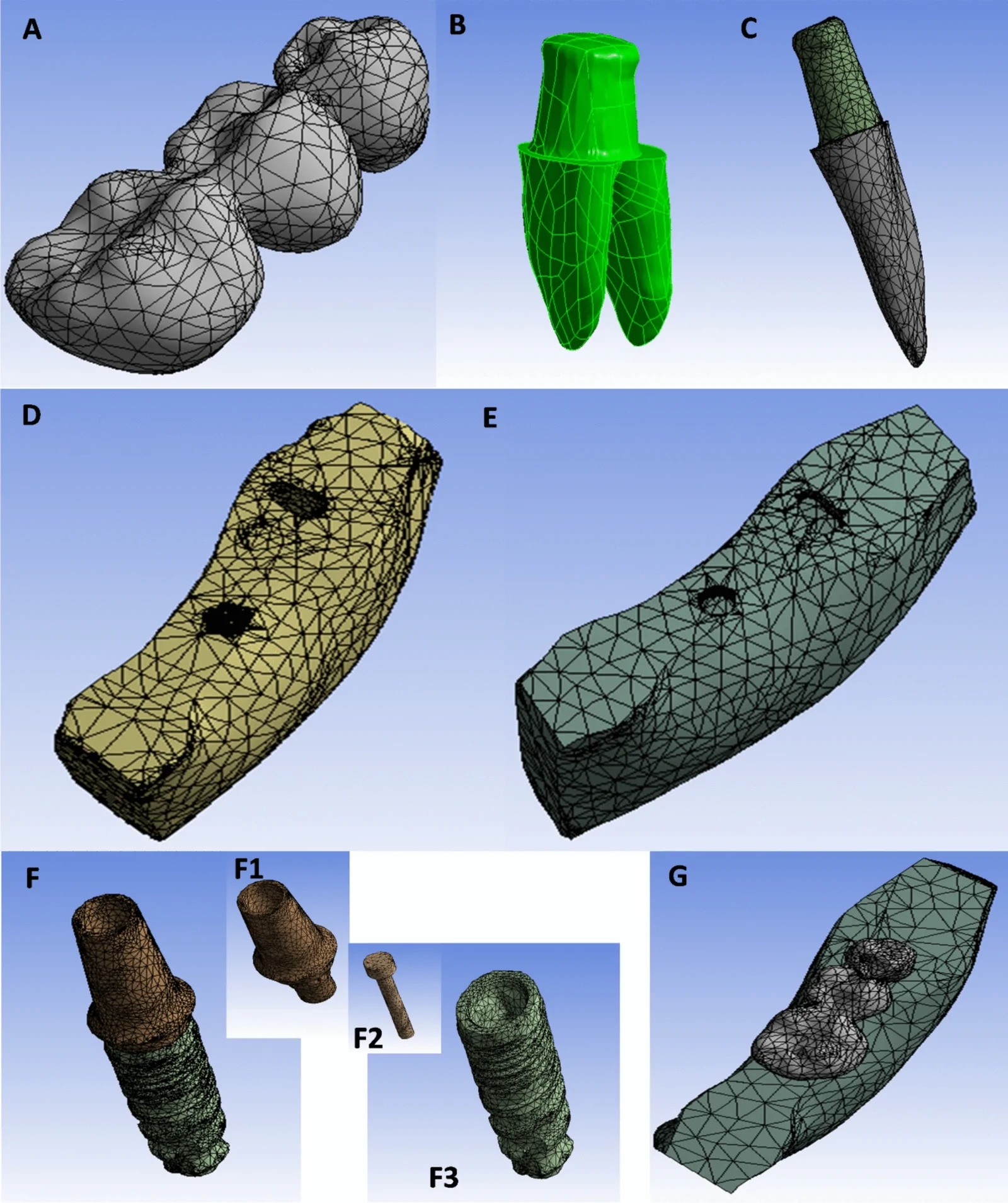
Components of the model: **A** three-units prosthesis, **B** molar tooth complex (prepared tooth and PDL), **C** premolar tooth complex (prepared tooth and PDL), **D** compact bone, **E** spongy bone, **F** implants complex (abutment (F1), screw (F2), and fixture (F3)), **G** meshed model

Solid models were exported as STEP files and imported into ANSYS Workbench 14.0 (ANSYS, Canonsburg, PA, USA) for meshing and simulation. Boolean operations were performed to validate all interfaces and ensure continuity across contact surfaces. Volume meshing was carried out using 3D solid elements (Element 187), suitable for irregular geometries. To ensure numerical reliability, a mesh convergence test was performed. Test loads were applied to multiple mesh densities, and convergence was defined by von Mises stress values in the cortical bone varying within ± 5%. A mesh growth rate of 1.2 and element sizes between 0.005 mm and 0.991 mm were employed to accommodate complex anatomical contours. Final element and node counts for each model are detailed in Table 1, and component meshes are illustrated in Fig. 1.

**Table 1:** Mesh density of the three models’ components

	Model 1: T4 + P5 + I6		Model 2: I4 + P5 + T6		Model 3: I4 + P5 + I6	
Part/material	Number of elements	Number of nodes	Number of elements	Number of nodes	Number of elements	Number of nodes
Prosthesis	24,623	39,966	38,361	57,897	38,826	78,796
Abutment	23,130	44,655	23,572	45,661	16,518	28,968
Screw	20,465	35,947	20,490	35,972	22,252	38,286
Implant	3,807	6,750	3,807	6,750	7,752	13,689
Tooth	27,959	48,078	27,862	47,918	–	–
PDL	23,670	47,023	24,996	49,925	–	–
Soft tissue	8,770	15,641	2,215	4,020	3,590	6,714
Cortical bone	9,146	16,214	8,322	15,870	7,256	13,464
Spongy bone	14,708	26,183	10,760	19,870	23,071	38,682

### Material properties and boundary conditions

All materials in the simulation were considered isotropic, homogenous, and linearly elastic. Material properties were obtained from validated literature sources (Table 2). Bonded contact conditions were applied at all interfaces to simulate complete osseointegration at the bone–implant interface and cold welding between implant components [23]. Boundary conditions were applied so that all nodes on the external surfaces of the bone block mesially and distally were fixed in all degrees of freedom. Before data extraction, validation of the geometric and loading models was confirmed by comparison with previously published studies [24].

**Table 2:** Materials properties used in the finite element models

Material	Modulus of elasticity in GPa	Poisson's ratio	References
Monolithic zirconia	200.00	0.26	Yousief et al. [25]
PEKK^a^	5.10	0.40	Alqurashi et al. [26]
Implant	110.00	0.34	De Moor et al. [27]
Tooth	18.60	0.31	Yousief et al. [25]
Periodontal ligament (PDL)	0.10	0.30	Elshazly et al. [28]
Mucosa	10.00	0.40	Abaza et al. [29]
Cortical bone	13.70	0.30	Al-Zordk et al. [24]
Cancellous bone	1.37	0.30	Al-Zordk et al. [24]

^a^Polyetherketoneketone

### Loading protocol and simulation parameters

Each model was subjected to a 50 N oblique static load applied at angles of 30° and 45° relative to the long axis of the second premolar. The loading site comprised ten predefined nodes at the functional cusp [20]. These angles were selected to replicate clinically relevant masticatory loads. Simulations were conducted using linear static analysis on an HP Z820 workstation (HP, Palo Alto, CA, USA) equipped with dual Intel Xeon E5-2660 processors (2.2 GHz) and 64 GB RAM. Output parameters included von Mises stress and total deformation values across the bone, prosthetic components, and supporting structures.

### Statistical analysis

Unlike in vitro studies, FEA simulations are deterministic, yielding consistent outcomes for identical boundary conditions and material properties. Hence, traditional statistical measures such as mean and standard deviation are not applicable. Instead, sensitivity analysis was used to evaluate the influence of mesh density on output precision, as detailed in the mesh convergence protocol, especially in all critical regions, including the implant–bone interface, fixation screw, and periodontal ligament [19].

## Results

**Fig. 2:**
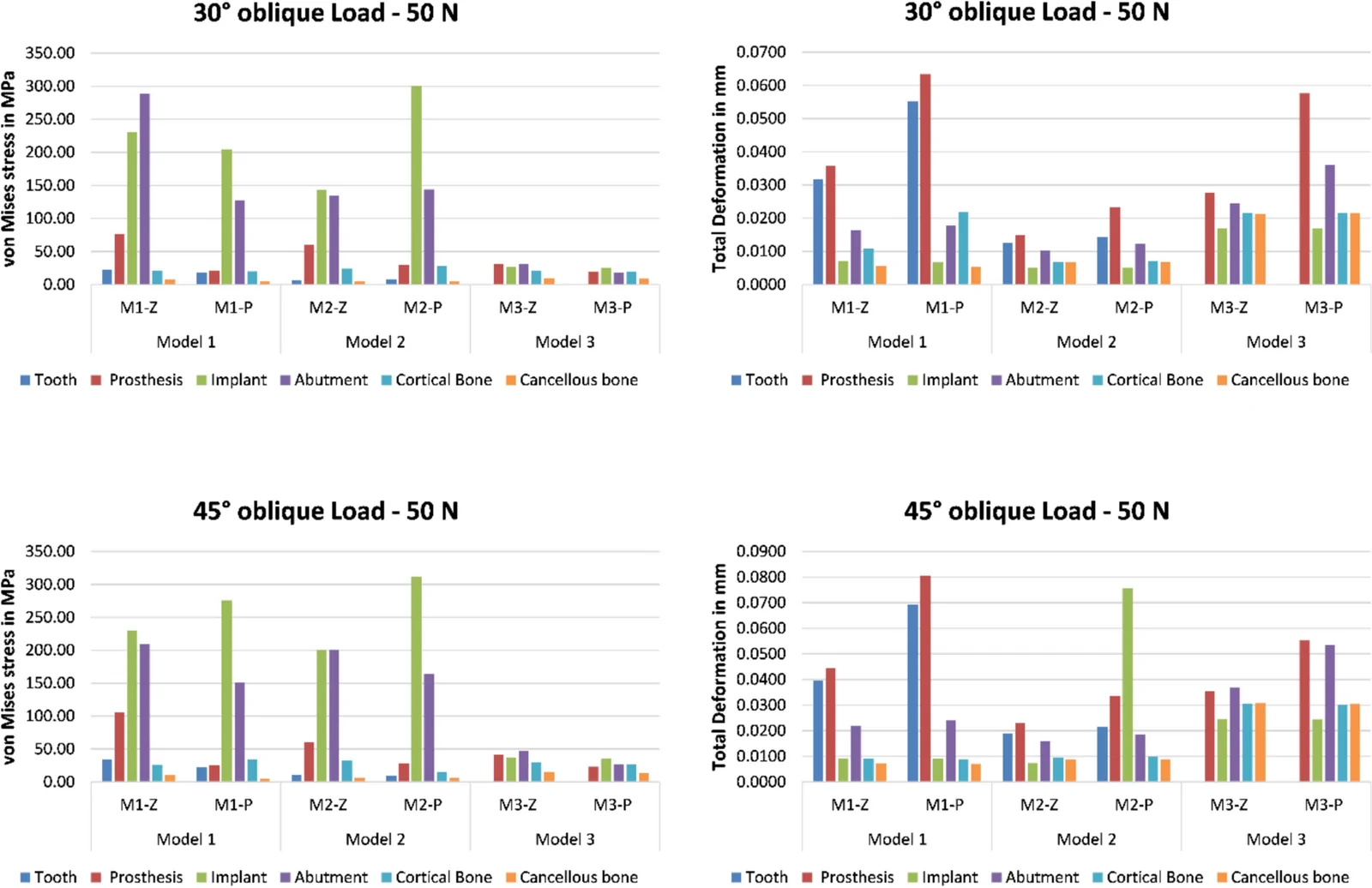
Comparison of equivalent von Mises stress (left) and total deformation (right) in the tooth, prosthesis, implant, abutment, cortical bone, and cancellous bone for six model configurations (M1-Zirconia, M1-PEKK, M2-Zirconia, M2-PEKK, M3-Zirconia, and M3-PEKK) under 30° (top) and 45° (bottom) oblique loading, illustrating the effects of design and material selection

Overall, the M3 configuration (implant–implant-supported) demonstrated the most favorable biomechanical performance, characterized by the lowest von Mises stress values across all components under both 30° and 45° oblique loading conditions (Fig. 2).

### Implant assembly

In comparison to M3, the M1 and M2 configurations exhibited higher von Mises stresses within the implants. The highest implant stress was recorded in M2 with PEKK (M2-P) under 45° loading, reaching 312 MPa, which was also associated with the greatest total deformation at 75 µm. The connecting screw experienced the highest stress in M1-P, reaching 483 MPa under the same loading condition. The highest stress in the implant abutment occurred at the occlusal margin, whereas the implant fixture showed its maximum stress at the crestal region at the bone–implant interface.

### Tooth and PDL

In both M1 and M2, stress values in the tooth and surrounding PDL were notably lower than in the implant components. However, M1 consistently exhibited greater stress and deformation in the tooth compared to M2 under both loading angles. Material comparison revealed that zirconia-supported prostheses generated higher stress within the tooth than PEKK-supported ones. The maximum stress was observed in M1-Z at 45°, reaching 34 MPa.

For the PDL, stress was again higher in M1 than in M2, irrespective of the restorative material. The peak PDL stress was recorded in M1-P (0.13 MPa). In addition, PEKK prostheses induced greater deformation in both the tooth and PDL compared to zirconia. The highest deformations were seen in M1-P at 45°, reaching 69 µm in the tooth and 46 µm in the PDL.

### Prosthetic superstructure

Zirconia-based restorations exhibited higher von Mises stress values across all configurations when compared to PEKK. The greatest stress was recorded in M1-Z at 45°, with a value of 105 MPa. In contrast, PEKK frameworks showed more deformation, with a maximum value of 80 µm in M1-P at 45°.

Stress concentration sites within the prosthetic structures varied among configurations. In M1, peak stress occurred at the implant abutment. In M2, it was concentrated at the connector between the central pontic and the tooth abutment. In M3, the pontic itself was the primary site of stress accumulation.

### Bone and mucosa

Cortical bone stress remained within a moderate range (≤ 35 MPa) across all models, with peak values occurring at the implant–bone interface near the pontic region. These values showed minimal differences between zirconia and PEKK prostheses.

Cancellous bone stresses were uniformly low (< 15 MPa) under all conditions.

The mucosal tissue remained biomechanically stable across all models, with no significant influence observed from either the prosthetic design or material type.

## Discussion

This study employed finite element analysis (FEA) to evaluate the biomechanical performance of three prosthetic models: two hybrid implant–tooth designs and one implant–implant-supported prosthesis, each fabricated with two different materials (Zirconia and PEKK). While the vertical loading scenario has been well characterized in previous work [30], the present study extends these findings by assessing oblique loading conditions, which are known to produce higher stress concentrations. An oblique load of 50 N was applied at 30° and 45° to the occlusal plane to simulate functional lateral forces during mastication. Although lower in magnitude than the vertical load (100 N), oblique forces generate substantial bending and shear stresses, allowing critical stress regions within the prostheses and supporting structures to be identified. Moreover, this low load level also avoids nonlinear complexities while preserving linear stress scaling, meaning that higher loads would proportionally increase stress values without altering the comparative trends. Furthermore, using this lower standardized load ensures consistency across models and isolates the effects of configuration and material properties, in line with physiologically relevant loading ranges reported in previous biomechanical studies [31, 32].

The outcomes were evaluated in terms of von Mises stress (maximum element values) and total deformation. Von Mises stress provides a single equivalent value that represents the multi-axial stress state and predicts material yielding or failure under complex loading. Total deformation reflects the overall displacement of the structure under load, indicating the extent of shape change or movement. In the context of tooth–implant- and implant–implant-supported prostheses, areas of elevated von Mises stress highlight regions at higher risk of material fatigue or fracture (bridges, connectors, or implants), whereas increased total deformation may indicate reduced prosthesis stability, altered occlusion, or greater micromotion at the implant–bone interface, potentially compromising long-term success [33].

The present FEA demonstrated that the implant–implant-supported configuration (M3) exhibited the most favorable biomechanical performance, consistently showing the lowest von Mises stress values across all components and loading conditions. In contrast, the tooth–implant-supported models (M1 and M2) showed increased stress concentrations, which can be attributed to the biomechanical mismatch between the mobile PDL-supported tooth and the osseointegrated, rigid implant. This mismatch creates a cantilever-like effect, concentrating stresses particularly around the implant components [34]. Moreover, the higher stress observed in M1 can be attributed to the longer functional cantilever created when the implant is placed distally, which increases bending moments on the natural tooth abutment. In contrast, mesial implant placement (M2) shortens this leverage effect, resulting in lower stress transfer to the tooth, particularly in zirconia-supported restorations. This aligns with previous work by Aboelfadl et al. [35], who reported that rigid, fixed–fixed implant-supported prostheses—especially those fabricated from high-stiffness materials such as zirconia—demonstrated more favorable stress distribution patterns with reduced implant and bone stress levels. However, it is important to note that Huang et al. [36] found no statistically significant clinical differences between tooth–implant and fully implant-supported prostheses regarding implant survival, prosthesis failure rates, technical complications, or marginal bone loss—indicating that with careful design, hybrid systems may remain clinically viable.

Oblique loading, as applied in this study, significantly increased stresses throughout the implant–prosthesis complex, affecting both the restorative components and surrounding bone. Prior FEA studies [37] have shown that oblique loading, particularly at 45°, can lead to elevated compressive stress in cortical bone on the side of implant inclination and tensile stress on the contralateral side, emphasizing the need to avoid unfavorable load directions in clinical practice. These biomechanical findings highlight the importance of reinforced prosthetic designs that can accommodate off-axis forces and reduce stress peaks under oblique loading conditions.

The peak screw stress in M1-P (483 MPa) under oblique load is consistent with evidence that oblique forces tend to load the abutment and screw more heavily, increasing risk for mechanical complications and screw loosening [38]. Similarly, Uppalapati et al. [39] found that implant abutments and screws experienced considerably higher von Mises stresses under oblique, rather than axial, loading, adding additional bending moments that intensified stress accumulation in these critical components. Moreover, several investigations [39–42] have confirmed that oblique loading shifts stress concentrations from the central implant body toward the implant neck and abutment interface, which increases mechanical demands on critical connector components and raises the risk of fatigue failure, micromotion, and eventual biomechanical breakdown over time.

Regarding material selection, zirconia demonstrated lower deformation but higher internal stress within the prosthetic framework, consistent with its high elastic modulus and load-bearing nature. This behavior corroborates previous studies [34, 35, 43], which suggested that although zirconia transmits higher internal stress, it distributes occlusal forces more efficiently to adjacent structures. In contrast, PEKK—a high-performance polymer with a lower elastic modulus—exhibited greater deformation in the prosthesis, natural tooth, and periodontal ligament (PDL), reflecting a more flexible behavior. These findings are consistent with prior research [35, 44, 45], which reports that high-elasticity polymers can reduce stress transfer to the tooth structure while being associated with increased deflection and a potential risk of structural compromise. PEKK, in particular, exhibits an elastic modulus closer to that of dentin, allowing for a more biomimetic stress distribution. Although PEKK demonstrated greater deformation than zirconia, the maximum displacement observed (75 µm) falls within ranges previously reported as clinically acceptable for elastic deformation in prosthetic systems. Nevertheless, this increased flexibility highlights the importance of optimized framework design and precise occlusal adjustment to limit excessive micromovement during long-term function [44].

Bone response across all configurations remained within physiological limits. Cortical bone stresses were moderate (≤ 35 MPa), and cancellous bone stresses were consistently low (< 15 MPa), regardless of prosthetic material or design. These outcomes suggest that all prosthesis types evaluated in this study are biomechanically compatible with bone tissue. This is supported by clinical and computational evidence [46–48], which shows that well-designed posterior prostheses—whether fully implant-supported or hybrid—can maintain physiological load transfer and support long-term bone homeostasis. However, models M1 and M2 exhibited approximately four to five times higher stresses on the implant complex compared with Model 3, indicating they represent critical or borderline cases for fatigue failure over time. The von Mises stresses in these models exceeded the typical fatigue (endurance) limit of titanium, generally considered to be 300–350 MPa [49]. For instance, a stress level of 483 MPa corresponds to an estimated fatigue life of roughly 600,000 loading cycles, indicating a substantially increased risk of screw loosening and fatigue fracture under repeated functional loading.

Despite the insights gained, this numerical study has some limitations. All structures were modeled as homogeneous, isotropic, and linearly elastic, which does not fully capture the heterogeneous, anisotropic, and viscoelastic properties of biological tissues in clinical reality. Furthermore, the simplified loading conditions do not reflect the dynamic and multifactorial forces encountered in the oral environment. Moreover, the assumption of perfect osseointegration, as simulated as bonded contacts, is a simplification of the clinical reality. To enhance clinical relevance and the generalizability of results, future research should incorporate more physiologically realistic material models and complex loading scenarios, validated through in vitro and in vivo investigations.

## Conclusion

Within the limitations of this FEA study, the following conclusions can be drawn:

1. Implant–implant-supported prostheses exhibited the most favorable biomechanical behavior under oblique loading, with the lowest von Mises stresses and minimal deformation, supporting their reliability in posterior restorations.
2. Tooth–implant-supported configurations, particularly with anterior implant placement (M2), showed increased stress and deformation, highlighting the influence of differential mobility and the potential for biomechanical complications.
3. Zirconia frameworks offered high rigidity and dimensional stability, resulting in greater internal stresses but reduced deformation, making them suitable for high-load applications.
4. PEKK frameworks, due to their lower stiffness, allowed greater deformation and better stress distribution to surrounding tissues. However, their increased flexibility necessitates precise prosthetic design to prevent excessive deflection.

## Clinical significance

This study highlights that implant–implant-supported prostheses appear to be the safest option under oblique loading, with lower stress and deformation and a reduced risk of mechanical complications. Tooth–implant-supported prostheses may still be used when implant placement is limited, but require careful occlusal adjustment and strategic implant positioning to minimize overload. Material choice is clinically relevant: zirconia provides higher rigidity and dimensional stability, while PEKK may help absorb functional forces but demands controlled occlusion and optimized framework design to prevent excessive deformation.

## Data Availability

Available on request.
